# Mechanisms shaping the gypsum stromatolite-like structures in the Salar de Llamara (Atacama Desert, Chile)

**DOI:** 10.1038/s41598-023-27666-5

**Published:** 2023-01-12

**Authors:** Joaquín Criado-Reyes, Fermín Otálora, Àngels Canals, Cristóbal Verdugo-Escamilla, Juan-Manuel García-Ruiz

**Affiliations:** 1 Laboratorio de Estudios Cristalográficos, IACT, UGR-CSIC, Av. Palmeras 4, 18100 Armilla, Granada Spain; 2 Departamento de Mineralogía, Petrología y Geología Aplicada, Facultad de Ciencias de La Tierra, Universidad de Barcelona, C/Martí i Franques s/n, 08028 Barcelona, Spain

**Keywords:** Planetary science, Chemistry

## Abstract

The explanation of the origin of microbialites and specifically stromatolitic structures is a problem of high relevance for decoding past sedimentary environments and deciphering the biogenicity of the oldest plausible remnants of life. We have investigated the morphogenesis of gypsum stromatolite-like structures currently growing in shallow ponds (*puquíos*) in the *Salar de Llamara* (Atacama Desert, Northern Chile). The crystal size, aspect ratio, and orientation distributions of gypsum crystals within the structures have been quantified and show indications for episodic nucleation and competitive growth of millimetric to centimetric selenite crystals into a radial, branched, and loosely cemented aggregate. The morphogenetical process is explained by the existence of a stable vertical salinity gradient in the ponds. Due to the non-linear dependency of gypsum solubility as a function of sodium chloride concentration, the salinity gradient produces undersaturated solutions, which dissolve gypsum crystals. This dissolution happens at a certain depth, narrowing the lower part of the structures, and producing their stromatolite-like morphology. We have tested this novel mechanism experimentally, simulating the effective dissolution of gypsum crystals in stratified ponds, thus providing a purely abiotic mechanism for these stromatolite-like structures.

## Introduction

Stromatolites are layered organo-sedimentary structures formed by sediment trapping, binding, and mineral precipitation within prostrate microbial communities termed algal mats^1–5^. These structures, more frequent in the past than nowadays, are commonly used as evidence of ancient microbial life and as environmental markers for the study of ancient shallow-water environments, especially in Archean and Proterozoic deposits, where they appear as one of the first forms of life on Earth^6^. The formation of stromatolites has been the subject of intense debate^5,7–9^ that is still open because: (a) the diversity of potentially organo-sedimentary structures (for instance, microbialites, stromatolites, or thrombolites), (b) the plausibility of producing them by abiotic mechanisms is claimed both in field studies^10,11^ and by numerical simulations^12,13^, and (c) despite the predominance of calcium carbonate stromatolites^14–16^, similar structures made of silica^17–22^ or gypsum^23–31^ have also been reported. The proper use of these structures as a proxy to past environments depends critically on our knowledge of the processes that shape stromatolites or structures that resemble them.

**Figure 1:**
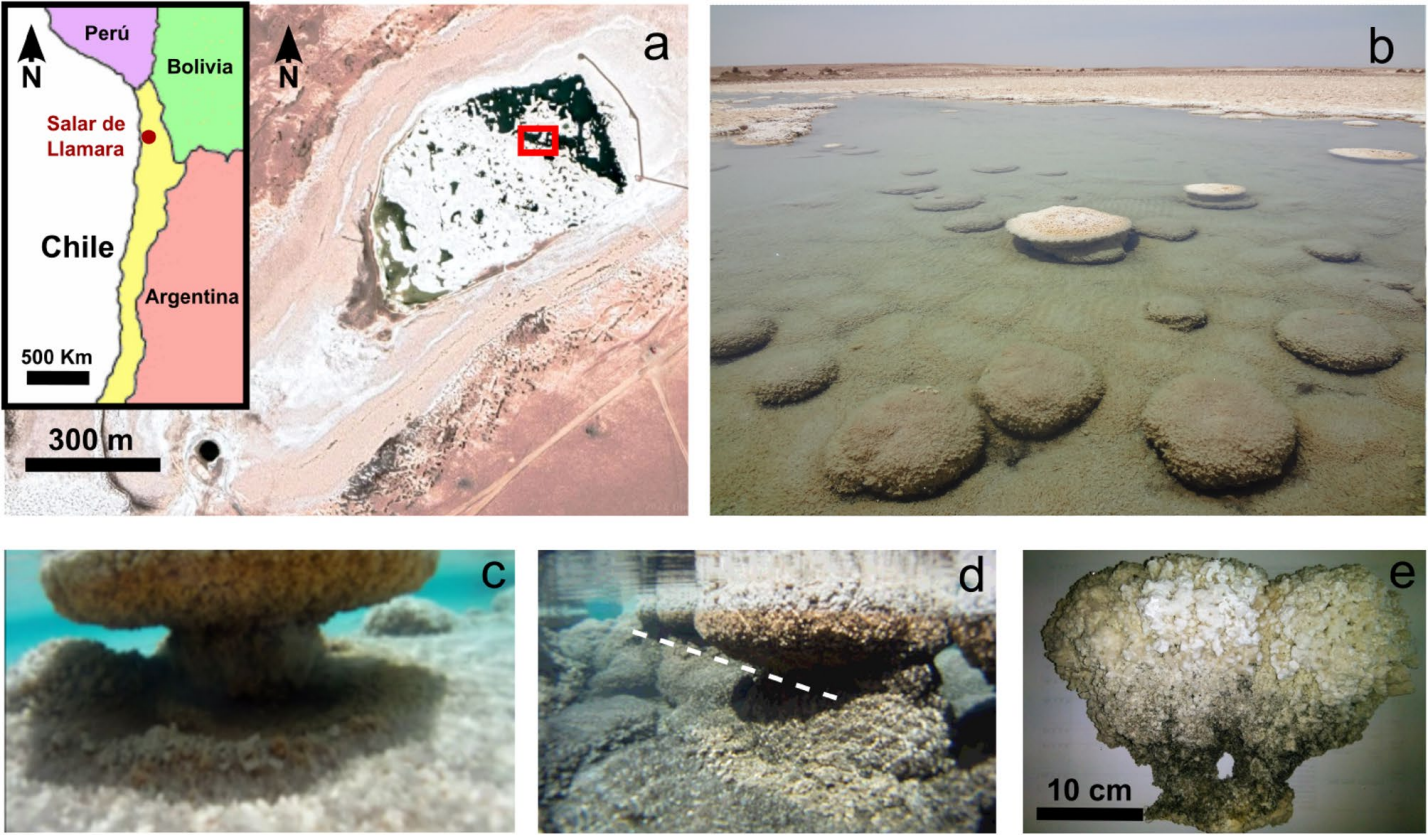
Gypsum stromatolite-like structures at the Huatacondo ponds locally named *puquíos*. (**a**) Location of the Salar de Llamara near the Pacific Coast of South America (inset) and Google Earth (2019) satellite image of the Puquíos de Huatacondo in the Llamara Salar, Atacama Desert, Chile. Red square is the location where photo (**b**) was taken. (**b**) Overall view of the gypsum stromatolite-like structures (GSLS) in a shallow *puquío*. (**c**) Lateral view of an in situ structure showing its characteristic mushroom shape and the accumulation of detrital gypsum particles under the GSLS. (**d**) In situ lateral view of a group of depth-aligned GSLS with narrow sections at the same depth (white dashed line). Detrital gypsum particles are also observed under the structures. (**e**) Lateral view of the studied GSLS (external surface). The size of GSLS shown in (**c**) and (**d**) are similar to the GSLS shown in (**e**). Additional images are available in Supplementary Figs. S1 and S2.

Gypsum microbialites, including stromatolites and thrombolites, have been reported in Messinian-age sediments outcropping around the Mediterranean Sea^32^ and are present in stratigraphic records of different ages in Ukraine, Australia, and Guatemala^33–35^. Different types of gypsum microbialites are contemporaneously forming in Egypt^27^, Saudi Arabia^36^, Venezuela^25^, and noticeably, because of their astrobiological relevance, at several locations of the Atacama Desert in northern Chile^29,37^. We have focused our investigation on the currently forming gypsum stromatolite-like structures (GSLS) in the Salar de Llamara (Tarapacá region, Pampa del Tamarugal, 141 km SE of Iquique, in the Atacama Desert). This basin hosts some large evaporite deposits (“salares”), produced by lacustrine-evaporitic sedimentation since the Miocene^38,39^, which represent reliable paleoclimatic and palaeohydrologic indicators^40,41^. “Salar” is the term used in South America and in this report for salt-encrusted playas. Similar landforms in the United States have been termed salt flats, or, less frequently, salt pans. The surface of the Salar de Llamara is a hard-saline crust made of recycled material from the Soledad Formation, which contains Pliocene halite and anhydrite deposits from ephemeral saline lakes, revealing a hydrological evolution from a saline pan to a salt-encrusted playa and, ultimately, to a gypsum-dominated playa lake^37,42,43^. In the Salar de Llamara, GSLS are currently forming in the hypersaline shallow ponds of Huatacondo, locally referred to as “*puquíos*” (Fig. 1a–e). The explanation of the morphogenesis of these structures is a challenge in mineral pattern formation with important applications for the detection of primitive life and the evaluation of past sedimentary environments. Using a combination of the previously studied complex hydrochemistry of the Puquíos de Huatacondo^37^, textural study techniques, and ad-hoc crystal growth/dissolution experiments, we propose an abiotic mechanism for the formation of the mushroom shape of GSLS.

## Results and discussion

### Characterization of the GSLS

**Figure 2:**
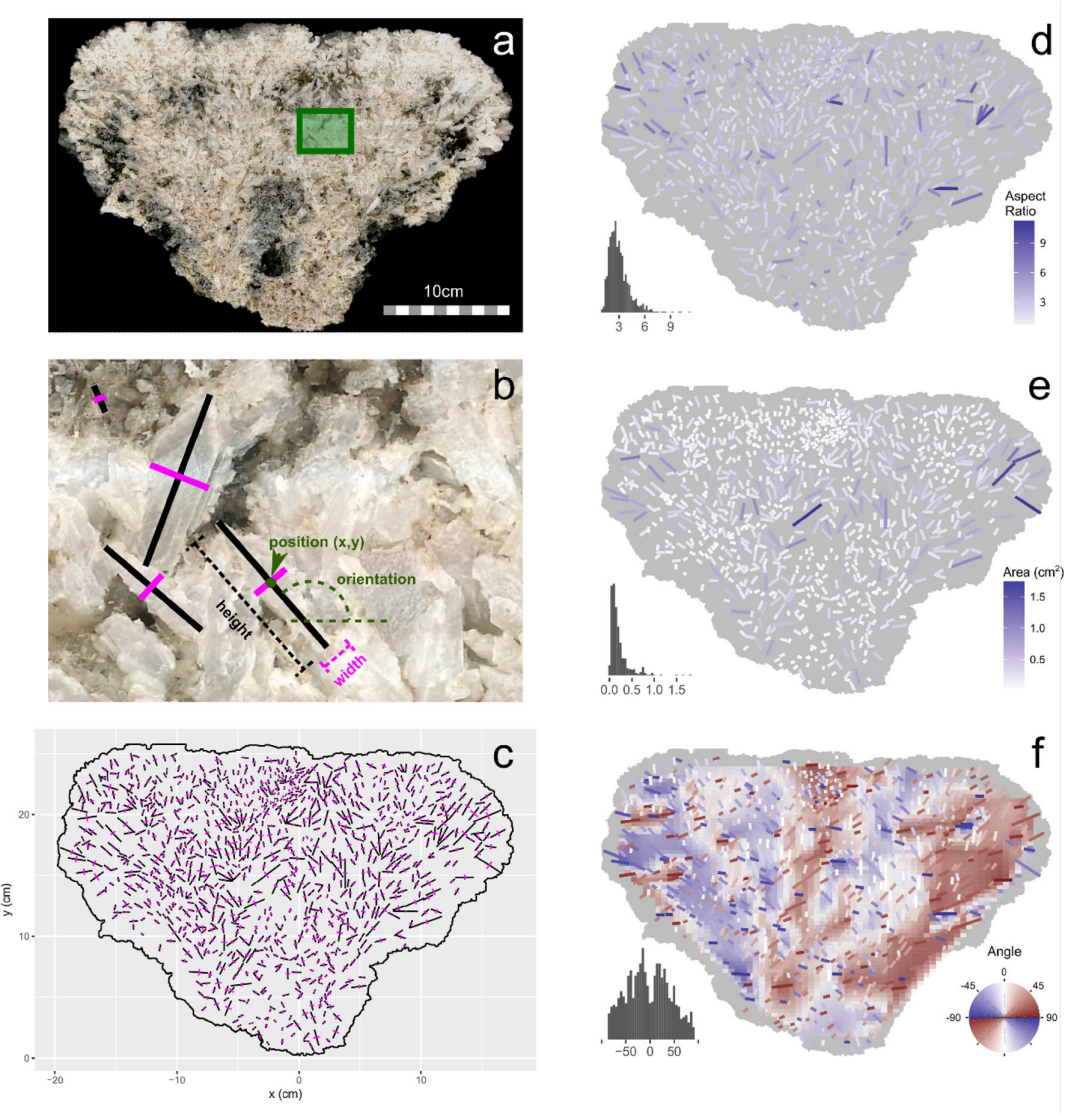
Quantitative characterization of the gypsum crystal distribution within the Gypsum Stromatolite-Like Structure. (**a**) Cross-section of the sample. Dark regions correspond to organic materials and/or large holes. (**b**) Detailed image (green rectangle in panel **a**) showing the crystals and the definition of parameters used in this study. (**c**) Long (black) and short (purple) segments of the measured crystals. (**d**) Aspect ratio distribution of the crystals, (**e**) Area distribution (cm^2^), (**f**) Orientation distribution (degree), the underlying colour map is an interpolation of the individual values. A histogram of the corresponding quantities is in the lower left corner of panels (**d**), (**e**) and (**f**).

GSLS forming in the Puquíos de Huatacondo (21° 16′ 06.5″ S 69° 37′ 02.1″ W) of the Salar de Llamara were investigated in situ during two field campaigns in October 2011 and March 2012 (Fig. 1a). The GSLS are presently forming in the eastern part of these *puquíos*. Accumulated detrital gypsum particles are found beneath the structure (Fig. 1c) and the alignment of structures in the same pond, with a narrowing at a common depth of around 20 cm was observed (Fig. 1d). We collected one GSLS during the most recent field campaign (Fig. 1e). That GSLS was supported by a thin stem that had recently broken, leaving the structure resting on one of its sides (Supplementary Figs. S1 and S2). We divided the GSLS radially into two halves. One of the halves was kept intact to observe the inner crystal distribution at the cutting surface (Fig. 2a). The second half was used to produce thin sections for textural studies using petrographic microscopy and for mineralogical characterisation by X-ray diffraction studies (Supplementary Table S1). X-Ray diffraction and optical microscopy show that the stromatolite is composed of gypsum while evaporitic minerals such as eugsterite, halite, and thenardite appear together along with gypsum, near the surface of the emerged part of the stromatolite. The submerged part is exclusively composed of gypsum.

Figure 2a shows a high-resolution scanned image (11,000 × 7300 pixels) of the cut surface used for crystal texture studies. The orientation, size, and shape of the crystals were automatically measured from manually defined pairs of orthogonal segments along the longest and shortest directions of the crystal (Fig. 2b). This measurement was repeated for 1007 different crystals distributed over the section and representing the majority of the population of crystals larger than 0.5 mm, which was limited by the image resolution of 34 microns per pixel (Fig. 2c and Supplementary Data S1). From these pairs of segments, we computed a set of “projected” morphological variables (Fig. 2b); the position of the crystal is defined as the intersection of the long and short axes, and the length and width correspond to the length of the long and short segments, respectively; we computed the aspect ratio as the ratio between length and width (Fig. 2d), size as the product of them, and orientation as the angle made by the longest direction with the horizontal. It must be stressed that these are "projected" or "apparent" values. This is unavoidable for the non-destructive characterization of many crystals. This was not an important limitation because the gypsum crystals are not randomly oriented with respect to the radial section; most of the crystals have c-axes oriented close to the section itself.

Crystals in the structure show a radial, branching distribution, already apparent in Fig. 2a, suggesting competitive growth limited by space availability. Crystal size (Fig. 2e) shows a continuous distribution (close to a negative exponential), cut at the smallest sizes because the measurement was limited to features larger than 10 pixels. Crystals of similar sizes do cluster, with large crystals aligning along the “stems” of the radial fans and around a horizontal plane about two-thirds from the base of the structure. This level likely corresponds to the water level during the later growth stages. The largest crystals are longer than 1.5 cm. The distribution of crystal aspect ratio values is between 2 and 3 and is relatively homogeneous within the structure (Fig. 2d).

Figure 2f visualizes the radial, fan-like distribution of crystals: crystal orientations deviating clockwise from the vertical are shown with a gradient from white (vertical) to red to dark grey (horizontal); crystal orientations deviating counterclockwise from the vertical are shown in white, blue, dark grey. The fan structure is therefore highlighted by the blue-white-red gradients in the underlying colour map. The orientation distribution is bimodal (Fig. 2f, histogram), with maxima at − 20° and + 20° corresponding to the left and right sides of each fan. The overall structure consists of two main fans with an opening of around 80° corresponding to twice the 40° separation between maxima. The skewed tails of the distribution correspond to the further bifurcation of the sub-fans. Notice that the left and right sides, in contact with the brine, show wider blue and red regions, respectively, more developed than the corresponding inner half fans. This feature is due to the availability of space to grow in that direction and confirms the competitive growth of the aggregate^44^.

**Figure 3:**
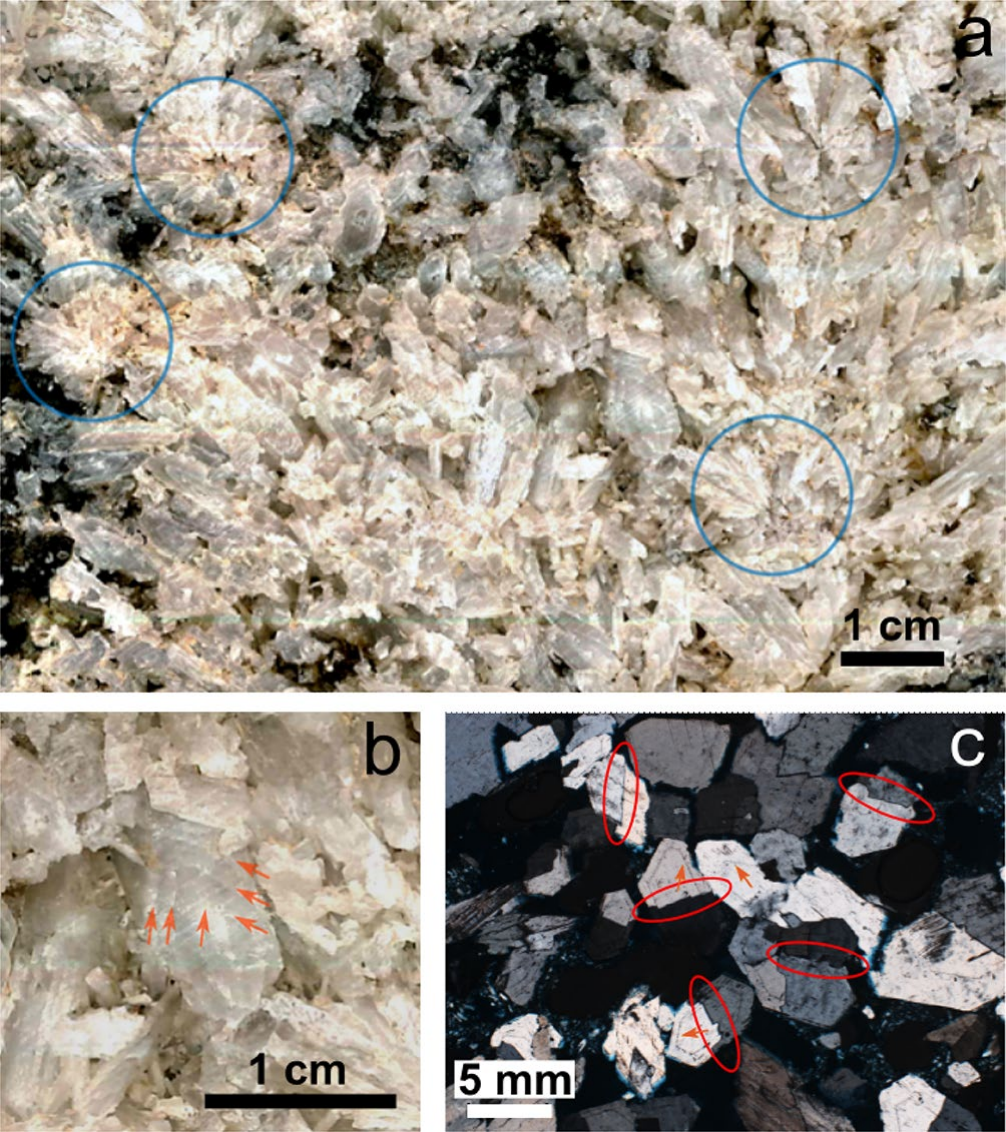
Crystal growth features displayed by crystals in the stromatolite-like structure. (**a**) Detail of the studied section of the stromatolite-like structure highlighting points of gypsum nucleation developing into local radial aggregates. (**b**) Gypsum crystal showing episodic growth surfaces (indicated by orange arrows). (**c**) Micrograph using polarized light of gypsum crystals showing the formation of (100) twin planes (indicated by red ellipsoids) and episodic growth surfaces (indicated by orange arrows).

We identified radial gypsum crystal aggregates (Fig. 3a), corresponding to high nucleation density events that may have been triggered by high supersaturation events or by the activity of cyanobacteria^6,18^. Our observations cannot confirm this biological effect, but certainly support the contribution of these radial aggregates to the branching and layering properties of the structure. The crystals which comprise the stromatolite were also studied by optical microscopy after embedding pieces cut from the sample in resin in order to mechanically stabilize them. This resin was dyed (blue) to reveal the pore space (Supplementary Fig. S3). Crystals often show recurrent episodic banded growth surfaces, with periods of alternation of relatively fast and slow growth (Fig. 3b,c), and are twinned following the twin law (100) (Fig. 3c). The distances between these growth bands are similar, which may be due to seasonal changes in brine composition.

All in all, our textural study suggests that the formation of these structures starts with the local nucleation of a few single crystals at the bottom of the pond. Competition for space during the growth of these crystals forced the development of a fan-like structure^37^. This structure develops by episodic nucleation of new fan-like aggregates and further growth of existing crystals. As a consequence, a hemispherical dome-shaped structure with a radial distribution of crystals is formed, a thrombolytic structure whose diameter can reach almost one meter. The question then remains, how does the narrowing of the GSLS occur? We find the answer to that question in the complex hydrochemistry of the *puquíos*.

### Gypsum growth/dissolution in stratified brines

**Figure 4:**
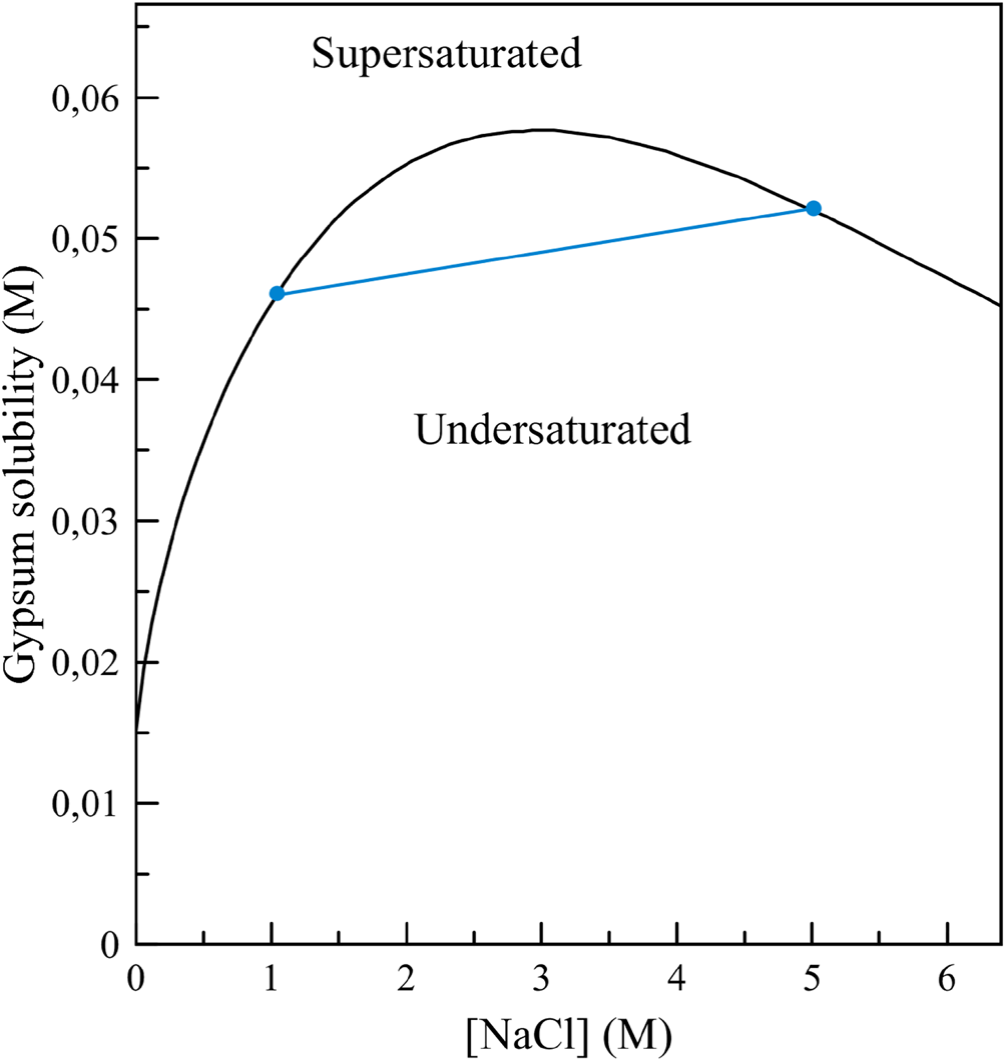
Solubility of gypsum as a function of NaCl concentration (calculated using the PHREEQC code and the Pitzer database). The compositions indicated by the two blue dots are those used in the experiment with synthetic solutions. The blue line represents the compositions obtained by mixing two brines equilibrated with gypsum.

The hydrochemistry of the Puquíos de Huatacondo in the Salar de Llamara is relatively complex and is very relevant for explaining the morphogenesis of GSLS^37^. In our previous paper, we have identified three compositional changes^37^, mainly related to the salinity of the brines, i.e., to the concentration in calcium sulphate and sodium chloride: (1) a “lateral” compositional gradient from west to east due to the progressive evaporation of the brines as they flow through the *puquíos*, (2) a vertical (depth) compositional gradient related to a density stratification of the brines, and (3) a temporal “seasonal” variation of brine composition. The lateral gradient is relevant for the formation of the GSLS since they only develop in the eastern end of the group of ponds, where salinity is the highest and algal mats are less developed^45–47^. The episodic growth of the crystals shown in the previous section is the consequence of the seasonal compositional variation of brine. Finally, the vertical stratification of the *puquíos* brines is the key factor, because it is relevant to the change of gypsum solubility as a function of NaCl concentration: As shown in Fig. 4, calculated using the hydrogeochemical software PHREEQC using the Pitzer database^48^, solubility is maximum in solutions with NaCl concentration of around 3 mol/L and decreases either if the concentration is reduced by dilution or increased by evaporation. The counterintuitive consequence of this maximum solubility is that by mixing two saturated gypsum solutions (for instance, see dots in Fig. 4), we obtain an undersaturated solution with respect to gypsum (any point on the blue line joining the two blue dots). The precipitation mechanism, or, dissolution of a phase by mixing solutions with non-linear solubility as a function of salinity, has been theoretically proposed^49^. Dissolution by brine mixing has been suggested in carbonates^50^. However, to our knowledge, the mechanism of gypsum dissolution by brine mixing in nature has never been reported.

Brines within the Puquíos de Huatacondo are density-stratified; both the top and bottom brines in a given pond are almost in equilibrium with gypsum^37^, the compositions in a vertical profile of the pond will fall on a line segment such as the one shown in Fig. 4. At intermediate depths, the composition of the brines will be undersaturated. Within the GSLS, crystals located close to this halocline would dissolve.

**Figure 5:**
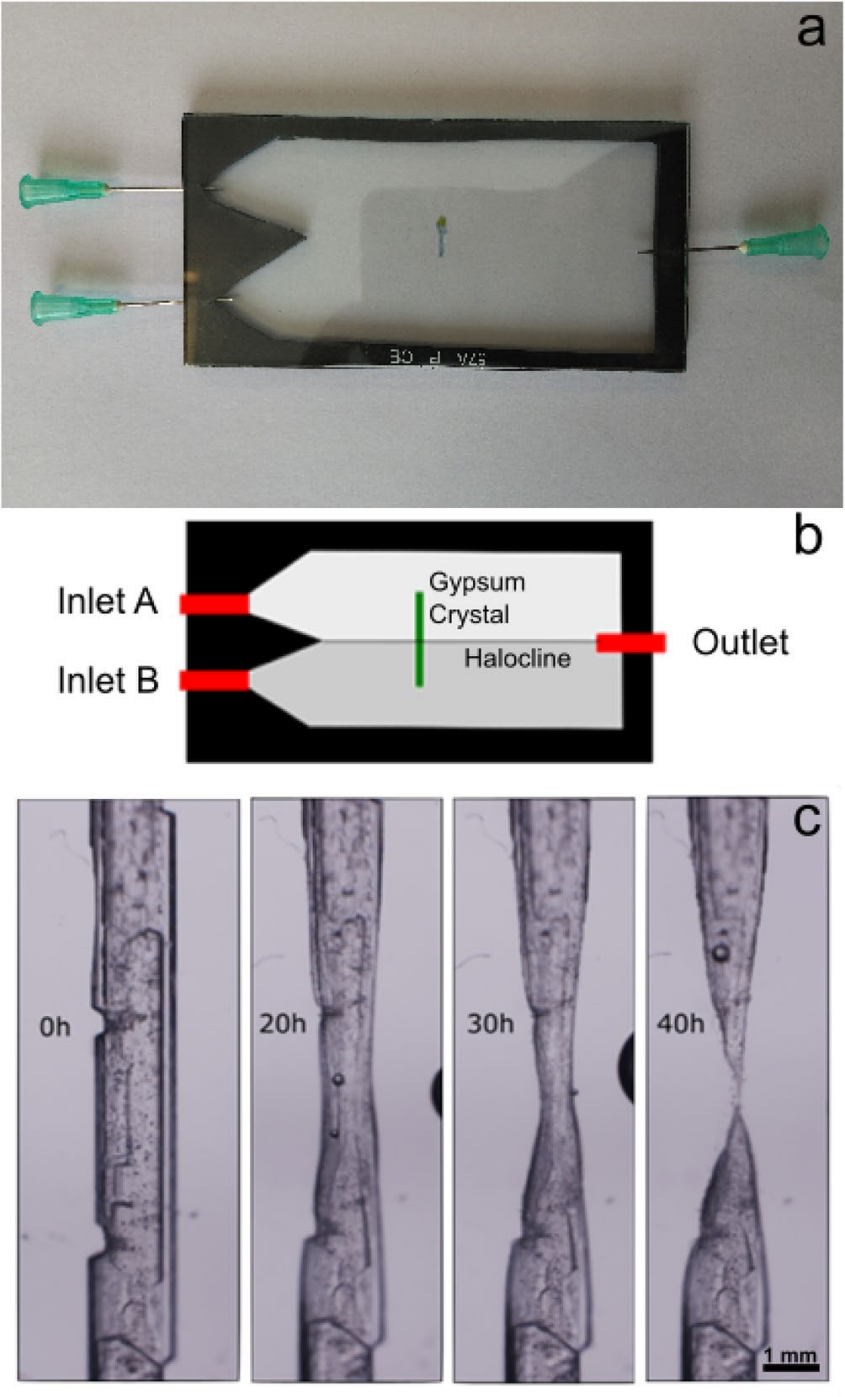
Actual view (**a**) and schematic representation (**b**) of the halocline flow setup. Inlets and outlet are marked in red; the rubber separator is shown in black. The green segment represents the position of the gypsum crystal inside the flow chamber. The white and grey areas represent the space filled with both solutions. (**c**) time-lapse images of the dissolution of the gypsum crystal at the halocline of the first experiment between solutions 1 M and 5 M of NaCl. The interface between both solutions is at the vertical centre of the images. The original video can be found in Supplementary Video S1.

To test this hypothesis, we have designed and performed ad-hoc experiments using a crystal growth cell that generates a permanent halocline (Fig. 5) that mimics the stratified brines of the Llamara ponds. This setup features a slow-flow chamber with two inlets at different heights and a single outlet (waste) in the centre of the opposite side. Two solutions, saturated with respect to gypsum but having different salinities, were slowly pumped from the left side to produce a laminar flow towards the right side, resulting in mixing by diffusion at the interface, thereby generating a steady halocline with a definite width depending on the residence time. Two NaCl solutions (1 M and 5 M) were equilibrated with gypsum for two weeks to obtain the composition of the blue dots in Fig. 4. The denser one (5 M NaCl) was pumped through the lower inlet and the lighter one through the upper inlet to keep the flow and mixing stable. One elongated gypsum crystal was perpendicularly fixed at the level of the halocline using a small droplet of wax. During the flow experiment, the central part of the crystal was observed under the microscope through a 45° mirror to keep the flow chamber vertical (Supplementary Fig. S4). Time-lapse images collected during the flow experiment show local crystal dissolution at the level of the halocline (Fig. 5c). After 40 h, the central part of most crystals was dissolved entirely, and the lower part of the crystals fell to the base of the chamber. We interpret these falling gypsum fragments to be the origin of the detrital gypsum accumulation under the structures (Fig. 1c,d).

**Figure 6:**
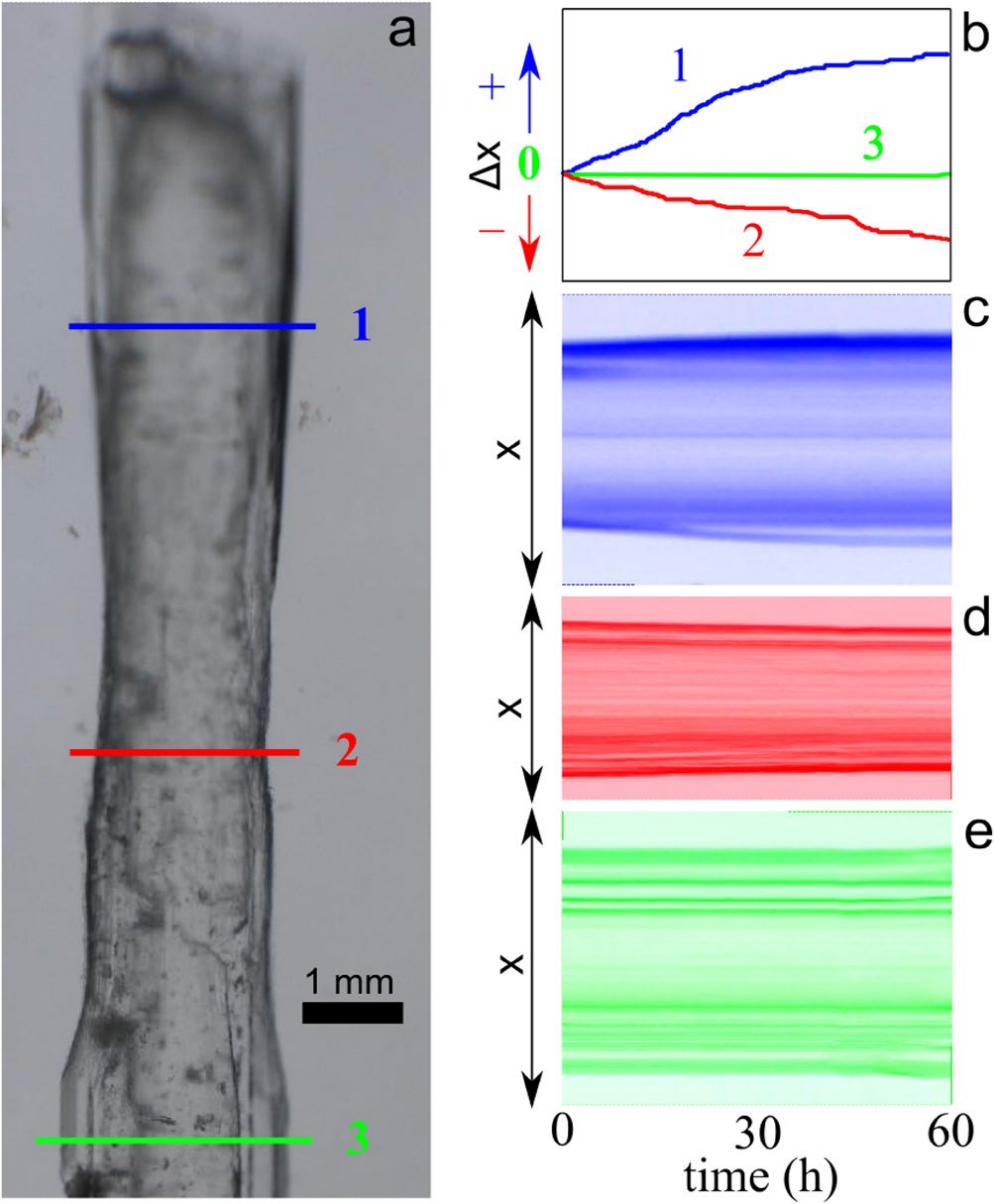
Natural brine mixing experiment. (**a**) Gypsum crystal after 60 h of stable halocline flow using natural brines from the top and bottom of the eastern pond. (**b**) Plot of the growth of the crystal at level 1, dissolution at level 2, and equilibrium at level 3. (**c**) Time-sliced image of 1, (**d**) time-sliced image of 2, (**e**) time-sliced image of 3. The original video can be found in Supplementary Video S2.

This experiment was performed using synthetic solutions with a high salinity contrast (4 M NaCl) to test, over a reasonable time scale, the plausibility of locally dissolving gypsum crystals in contact with a mix of two saturated solutions having different salinities. However, the salinity contrast between the top and bottom levels of the stratified *puquíos* is not that large (around 0.6 M). Hence, in a second run of experiments, we used natural brines sampled from Puquíos de Huatacondo (red square in Fig. 1a). We selected two brines from the same pond (Fig. 1b), one from the bottom of the pond (P12-9b), which is the most saline, and the other (less saline) from the top (P12-9)^37^. The brines were pumped through the chamber using the same setup and flow conditions of previous experiments (see “Methods”) and images were collected using the same protocol. Figure 6a shows the result of this experiment after 60 h. The narrowing of the central part is evident but, due to the lower salinity contrast, and therefore lower undersaturation at the halocline, the local growth/dissolution kinetics is much slower, and therefore, quantitative measurement (Fig. 6b) and time-slicing images (Fig. 6c–e) are required to properly assess the changes in crystal width at levels 1, 2 and 3 during the experiment. Different behaviours were found at these levels. At the top level (1), in contact with the lower salinity solution, the crystal grew slowly, while at the bottom level (3), in contact with the more saline solution, the width of the crystal remained constant during the experiment. At the level of the mixing region (2), we observed crystal dissolution, as expected, due to the undersaturation at the interface between both brines. A similar behaviour—neither growth nor dissolution—could be expected for levels 1 and 3, but in this experiment, the solutions used were sampled from the top and bottom of the *puquío* and full equilibrium with gypsum cannot be assumed for the top solution, where active evaporation was taking place at the time of sampling. This produces a small supersaturation with respect to gypsum in this solution, thus explaining the observed slow growth rate.

The above experimental results simply entail that density-stratified pond brines, which are close to equilibrium with respect to gypsum, but with higher salinity at the bottom, will be slightly undersaturated below a given depth. This will produce a dissolution and consequently a narrowing of the middle-lower part of the GSLS, explaining its mushroom shape.

Previous articles have reported that biological activity can influence gypsum precipitation, either at the stage of nucleation or growth^31,45^. Gypsum stromatolites in other Atacama *salares* have been proposed to result from biological and abiotic processes^29^. However, the structures reported in this work are rather gas-triggered and develop by successive mat growth, upholstery of the mat, formation of the gypsum cover, and crystal growth. Still, in this model, the development of the mats is not related to gypsum precipitation and the morphology of the structures (bubble-like, gas-filled domes covered with a single palisade of elongated gypsum crystals) is entirely different from the mushroom-like morphology of the GSLS found in the Puquíos de Huatacondo. We have noticed the presence of organic matter remains around the stem and in the holes of the GSLS at the Salar de Llamara (Fig. 1e), but not on the surface. This scarcity of mats along with the isotopic data previously published^37^ suggests a slight relevance of biologically driven mechanisms in the control of the textural arrangements of the crystals. Moreover, there are certainly no signs of the role of biological processes in forming the shape of the GSLS.

## Conclusions

GSLS currently forming in the *Salar de Llamara* are mushroom-shaped agglomerates composed of millimetre- to centimetre-sized gypsum crystals. The gypsum crystals show episodic growth and are arranged in radially distributed branching fans. The crystal texture and morphology of the structures are controlled by the three chemical gradients identified at the Puquíos de Huatacondo; the lateral gradient is responsible for the fast, competitive growth of gypsum that produces the GSLS only in the ponds containing the most concentrated brines. The seasonal variations in the composition and level of the brines produce further spherulitic nucleation and competitive crystal growth leading to the formation of hemispherical dome-shaped thrombolytic structures with a radial distribution of crystals. The mushroom shape of the GSLS is formed because of the selective dissolution of gypsum controlled by a gradient, namely, the stratification of highly concentrated brines that produce a level of undersaturated solution at the halocline between the lighter (top) brine and the denser (bottom) brine. The gypsum solubility dependence on brine salinity (Fig. 4), along with the density stratification, creates gypsum undersaturation at the halocline, progressively dissolving the lower part of the thrombolytic structure and thus explaining the present-day mushroom shape. The rate of the resultant dissolution is proportional to the salinity contrast between the upper and lower brines and is faster in the more evaporated eastern *puquíos*, where the GSLS are found.

Deciphering the biological or abiotic origin of biomimetic structures is a hot topic in the science of pattern formation because of their importance in primitive life detection on Early Earth and elsewhere^51,52^. Carbonated stromatolite structures, while they are not fossils sensu stricto, are considered the oldest known remnants of biological activity. However, the formation of similar structures by completely inorganic mechanisms has also been reported^12^ thus creating a controversial matter of discussion^53^. So far, the discussion has been focused on carbonate stromatolites, the most abundant on Earth. Deciphering the possible mechanisms of forming stromatolite structures in gypsum is also very important for astrobiological studies. Large gypsum deposits have been reported on Mars^54^, and permanent hypersaline water bodies have been discussed in the context of life-search initiatives^55^. Since our genetic mechanism is based on simple physicochemical premises, it applies to many different geological environments, past or present, and non-terrestrial environments. Interestingly, it is applicable either in the presence or absence of life.

## Materials and methods

We obtained the mineralogical composition of the samples by powder X-ray diffraction using dry samples from different parts of the GSLS after grinding manually with a mortar. The composition was assessed by quantitative phase analysis using Rietveld methods implemented in the TOPAS Academic V.5 software. The diffraction experiments were carried out using a Bruker, AXS D8 Advance Vario diffractometer (Cu Kα1) equipped with a Lynxeye detector and a primary germanium monochromator. Measurements span a 2θ angular range of 10°–100° with a 2θ step width of 0.015°.

Morphometric analysis of crystal distribution was done using custom Python 3 scripts to extract the position of all segments from an SVG overlay on top of the digitized image. Segments on this overlay were defined using the software Inkscape version 1.1.

Hydrochemical calculations of solubility and supersaturation were performed using the PHREEQC 3.4 code and the accompanying Pitzer database^48^.

For the first gypsum dissolution experiments (Fig. 5), the solution injected through inlet A was NaCl 1 M and the solution injected through inlet B was NaCl 5 M (both prepared from Sigma Aldrich 99% NaCl and MiliQ water type I). 2 g of CaSO_4_·2H_2_O powder (Sigma-Aldrich 98%) was set in contact with the solutions for two weeks under stirring to equilibrate them with respect to gypsum. After this period, solutions were filtered and stored in sealed bottles.

The halocline flow setup was implemented using two 10 × 5 cm glass plates separated by a 1 mm-thick rubber spacer (Fig. 5). Two liquid inlets and one liquid outlet (waste) were implemented by inserting syringe needles in the two opposite short sides. Pumping through the inlets creates an interphase layer between two solutions with different salinities. To avoid turbulent mixing, the flow rate was set to 1 mL/min and the setup was kept vertical and steady with the denser solution at the bottom. Natural, elongated gypsum crystals obtained from the evaporation of Huatacondo's brines were glued to one of the glass plates so that their centre lay at the halocline level.

Time-lapse microscopic observations were performed with a Nikon AZ100 microscope equipped with a Nikon AZ Plan Flour 2 × objective and a Nikon DS-Fi1 photographic camera. Pictures were acquired and analysed using the NIS-Elements BR software.

## Supplementary Information

Supplementary Information 1.

Supplementary Information 2.

Supplementary Video 1.

Supplementary Video 2.

## Data Availability

All data generated or analysed during this study are included in this published article and its supplementary information files.
