# Supplementary material for: Mechanisms shaping the gypsum stromatolite-like structures in the Salar de Llamara (Atacama Desert, Chile)
Source: Sci Rep. 2023 Jan 12;13:678. doi: 10.1038/s41598-023-27666-5 (PMC9837060; doi:10.1038/s41598-023-27666-5)
Supplement: Supplementary file 2 — Supplementary Information 2. [file 41598_2023_27666_MOESM2_ESM.docx]

Mechanisms shaping the gypsum stromatolite-like structures in the Salar de Llamara (Atacama, Chile). Supplementary Material

Joaquín Criado-Reyes^1^, Fermín Otálora^1^, Àngels Canals^2^, Cristóbal Verdugo-Escamilla^1^, and Juan Manuel García-Ruiz^1^


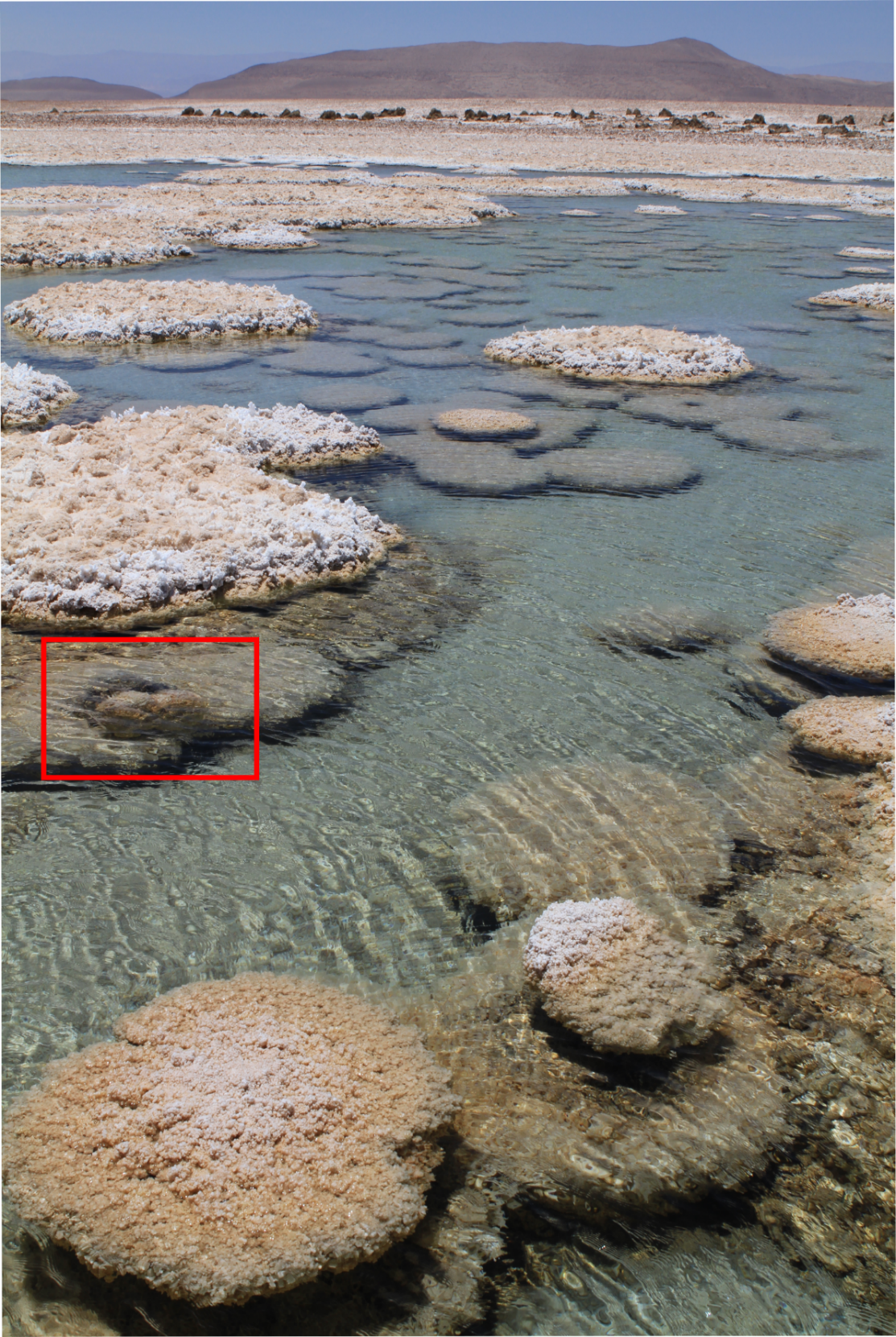


**Figure S1.** Photography of the emplacement of the fallen stromatolite used in our study (red square).


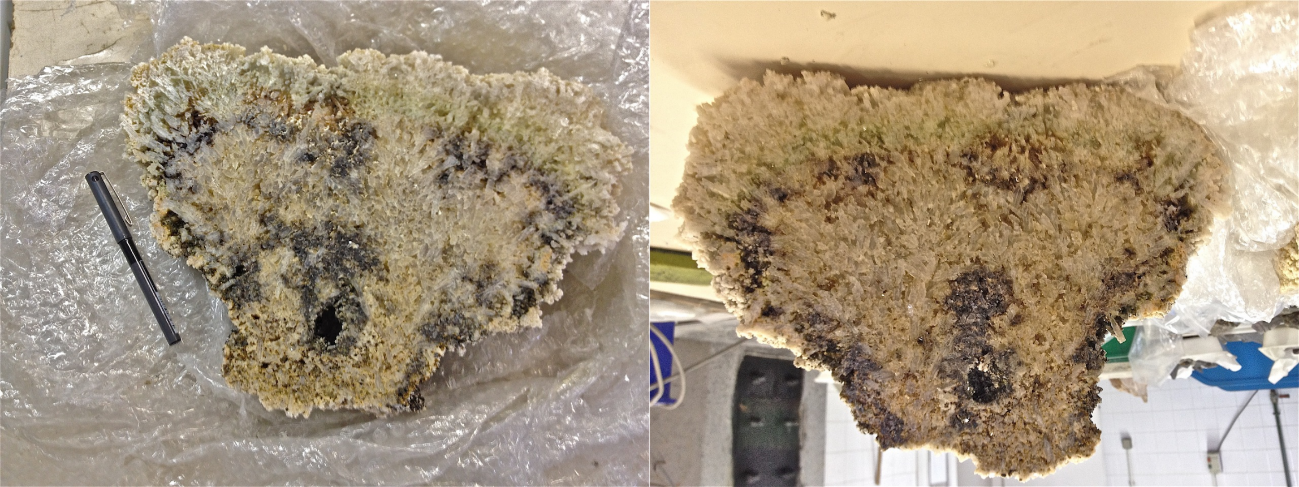


**Figure S2.** The two halves of the stromatolite collected.


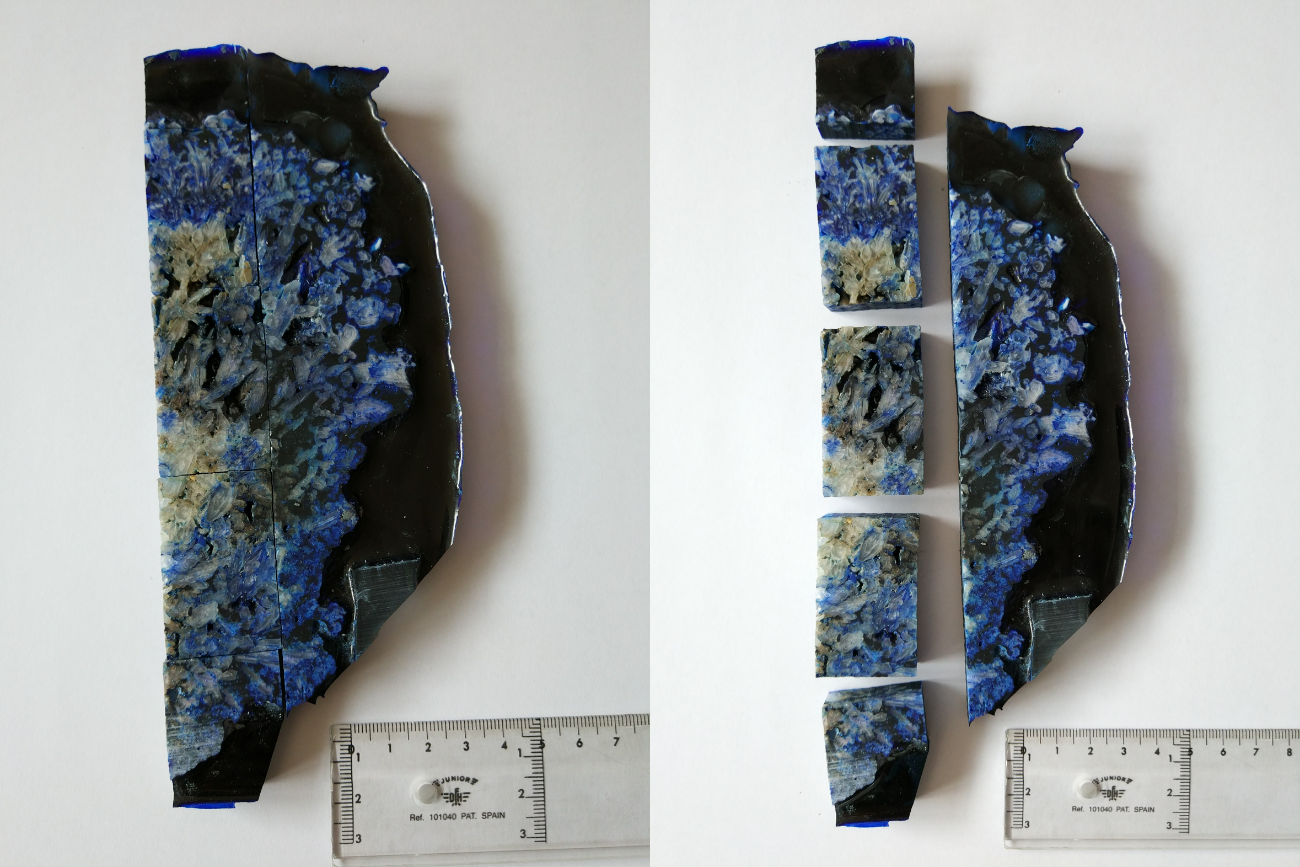


**Figure S3.** Part of the stromatolite embedded in blue resin (left) and the cut slices from it (right).


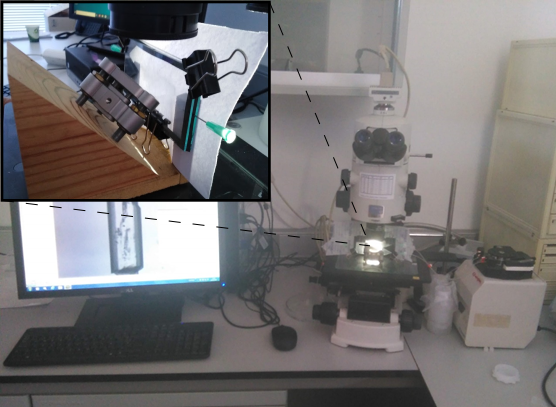
**Figure S4.** Permanent Halocline Device (PHD) places vertically under the microscope. A mirror with an inclination of 45º is placed in front of PHD to change the light pathway of the microscope from the vertical plane to the horizontal plane. PHD was monitored with a CCD camera. Pump and solutions are located to the right of the microscope, and waste is located to the left of the microscope.

**Table S1.** X-ray analysis of powder samples collected at different places of a gypsum mushroom structure from Huatacondo`s lakes.

| **Sample Position on stromatolite** | Top (emerged part) | | | Top-mid | | | Bottom-mid | | | Bottom | | |
| --- | --- | --- | --- | --- | --- | --- | --- | --- | --- | --- | --- | --- |
| **Sample** | XRD11 | XRD12 | XRD13 | XRD21 | XRD22 | XRD23 | XRD31 | XRD32 | XRD33 | XRD41 | XRD42 | XRD43 |
| **Composition** | Gyp, Hal, Eug, The | Gyp, Hal, Eug, The | Gyp, Hal, Eug, The | 98% Gyp, 2%Hal | 99% Gyp, 1%Hal | 98% Gyp, 2%Hal | 97% Gyp, 3%Hal | 97% Gyp, 3%Hal | 97% Gyp, 3%Hal | 94% Gyp, 6%Hal | 97% Gyp, 3%Hal | 93% Gyp, 7%Hal |

* Hal = Halite, Gyp = Gypsum, The = Thenardite, Eug = Eugsterite

**Dataset S1 (Supplementary Material file).** Dataset of the position, width, length, and orientation of the gypsum crystals outcropping in the cross section of the stromatolite used to build the figure 2 of the main text.

**Movie S1 (Supplementary Material file).** Movie of the dissolution experiment carried out with the laboratory made solutions corresponding with figure 5 of the main text.

**Movie S2 (Supplementary Material file).** Movie of the dissolution experiment carried out with the natural brine solutions corresponding with figure 6 of the main text.
